# The role of phytomelatonin receptor 1-mediated signaling in plant growth and stress response

**DOI:** 10.3389/fpls.2023.1142753

**Published:** 2023-03-10

**Authors:** Dawood Khan, Nan Cai, Weilin Zhu, Leilin Li, Miao Guan, Xiaojun Pu, Qi Chen

**Affiliations:** Faculty of Life Science and Technology, Kunming University of Science and Technology, Kunming, China

**Keywords:** phytomelatonin, PMTR1, growing development, stress response, senescence

## Abstract

Phytomelatonin is a pleiotropic signaling molecule that regulates plant growth, development, and stress response. In plant cells, phytomelatonin is synthesized from tryptophan *via* several consecutive steps that are catalyzed by tryptophan decarboxylase (TDC), tryptamine 5-hydroxylase (T5H), serotonin *N*-acyltransferase (SNAT), and *N*-acetylserotonin methyltransferase (ASMT) and/or caffeic acid-3-*O*-methyltransferase (COMT). Recently, the identification of the phytomelatonin receptor PMTR1 in *Arabidopsis* has been considered a turning point in plant research, with the function and signal of phytomelatonin emerging as a receptor-based regulatory strategy. In addition, PMTR1 homologs have been identified in several plant species and have been found to regulate seed germination and seedling growth, stomatal closure, leaf senescence, and several stress responses. In this article, we review the recent evidence in our understanding of the PMTR1-mediated regulatory pathways in phytomelatonin signaling under environmental stimuli. Based on structural comparison of the melatonin receptor 1 (MT1) in human and PMTR1 homologs, we propose that the similarity in the three-dimensional structure of the melatonin receptors probably represents a convergent evolution of melatonin recognition in different species.

## Introduction

Melatonin (*N*-acetyl-5-methoxytryptamine) is an evolutionarily conserved chemical molecule that was first discovered in the bovine pineal glands in 1958 (Lerner et al., 1958). In mammals, it acts as a hormone that has a variety of physiological impacts, including effects on the circadian rhythm, sleep, the immune system, mood, body temperature, exercise, and food intake. Historically, the first melatonin receptor in animals (Mel1a) was cloned from *Xenopus laevis* in 1994 (Ebisawa et al., 1994). Mel1a is categorized into the G-protein-coupled receptor (GPCR) superfamily with seven transmembrane helices that respond and transduce extracellular stimuli into intracellular signals (Ng et al., 2017). To date, two melatonin receptors, MT1 (Mel1a) and MT2 (Mel1b), have been reported in mammals (Ebisawa et al., 1994; Reppert et al., 1995), and their structures have also been determined with X-ray crystallography (Johansson et al., 2019; Stauch et al., 2019).

Phytomelatonin, which refers to melatonin of plant origin, was first detected in 1995 by several independent research groups (Dubbels et al., 1995; Hattori et al., 1995; Vantassel et al., 1995). Subsequent studies showed that phytomelatonin is ubiquitous and present in all plant taxa from algae to angiosperms and that it regulates growth and development, seed germination, root growth, flowering and fruiting, senescence, and photosynthesis (Sun et al., 2021; Zhao et al., 2021c; Chen et al., 2022). In addition, it also plays a significant role in the response of plants to various biotic and abiotic stresses (Arnao and Hernández-Ruiz, 2014; Zhao et al., 2021a; Huang et al., 2022; Mushtaq et al., 2022; Khan et al., 2022a). In 2018, the first putative phytomelatonin receptor, AtPMTR1 (Phytomelatonin Receptor 1), was identified in *Arabidopsis thaliana* (Wei et al., 2018), which is a candidate GPCR-like protein that could directly interact with the G protein α subunit, GPA1, in *Arabidopsis* (Wei et al., 2018). More recently, it has also been found that *Arabidopsis* uses AtPMTR1-mediated phytomelatonin signaling mechanisms to regulate the circadian stomatal rhythm and stomatal immunity *via* GPA1/NADPH oxidase-mediated reactive oxygen species (ROS) signaling or *via* mitogen-activated protein kinase (MAPK) cascades (Yang et al., 2021).

Several PMTR1 homologs have been recently identified by several independent research groups. Together, these homologs play important roles in phytomelatonin signaling for seedling growth, leaf senescence, and plant innate immunity, as well as drought and salt stress tolerance (Yang et al., 2021; Wang et al., 2021b; Bai et al., 2022). In order to better understand the basic functions of phytomelatonin and PMTR1, we provide a critical analysis of the current relevant publications to review and highlight the regulation of phytomelatonin in plant stress resistance using PMTR1-dependent strategies. In addition, we discuss the structural similarity between the melatonin receptors from mammals and plants by combining the three-dimensional structures of the melatonin receptors obtained from the protein database banks or from AlphaFold predictions with those from experimental results that involved recently identified AtPMTR1 homologs. Ultimately, this review discusses novel research on PMTR1 receptors and proposes that their function is likely conserved in different plant species.

## Metabolism of phytomelatonin

Melatonin is an ancient and a conserved molecule (Tan et al., 2014). The synthesis of melatonin in vertebrates occurs mainly within the mitochondria (Tan and Reiter, 2019) and comprises four sequential steps: hydroxylation, decarboxylation, acetylation, and methylation. This process starts with the hydroxylation of tryptophan, which is catalyzed by tryptophan hydroxylase (TPH) to form 5-hydroxytryptophan. Sequentially, 5-hydroxytryptophan is then decarboxylated to produce 5-hydroxytryptamine (serotonin, 5-HT). Following one acetylation step and one methylation step, 5-HT is then converted into melatonin (Arnao and Hernández-Ruiz, 2014; Zhao et al., 2019a).

Following its formation, the melatonin produced in the mitochondria binds to the MT1 receptors localized on the outer mitochondrial membrane, which leads to the activation of mitochondrial MT1/G protein signaling and the inhibition of stress-mediated cytochrome *c* release that blocks caspase activation (Suofu et al., 2017). Given that the mitochondria are the major organelles that produce ROS and RNS (reactive nitrogen species) in the cell, melatonin plays a dual role as an antioxidant to diminish excess ROS and/or RNS and to mediate GPCR signaling to block cytochrome *c* release (Suofu et al., 2017). This automitocrine signaling mechanism allows for both the synthesis and signaling of specific ligands and the precise control of organelle-based intracellular signaling (Suofu et al., 2017).

**Figure 1:**
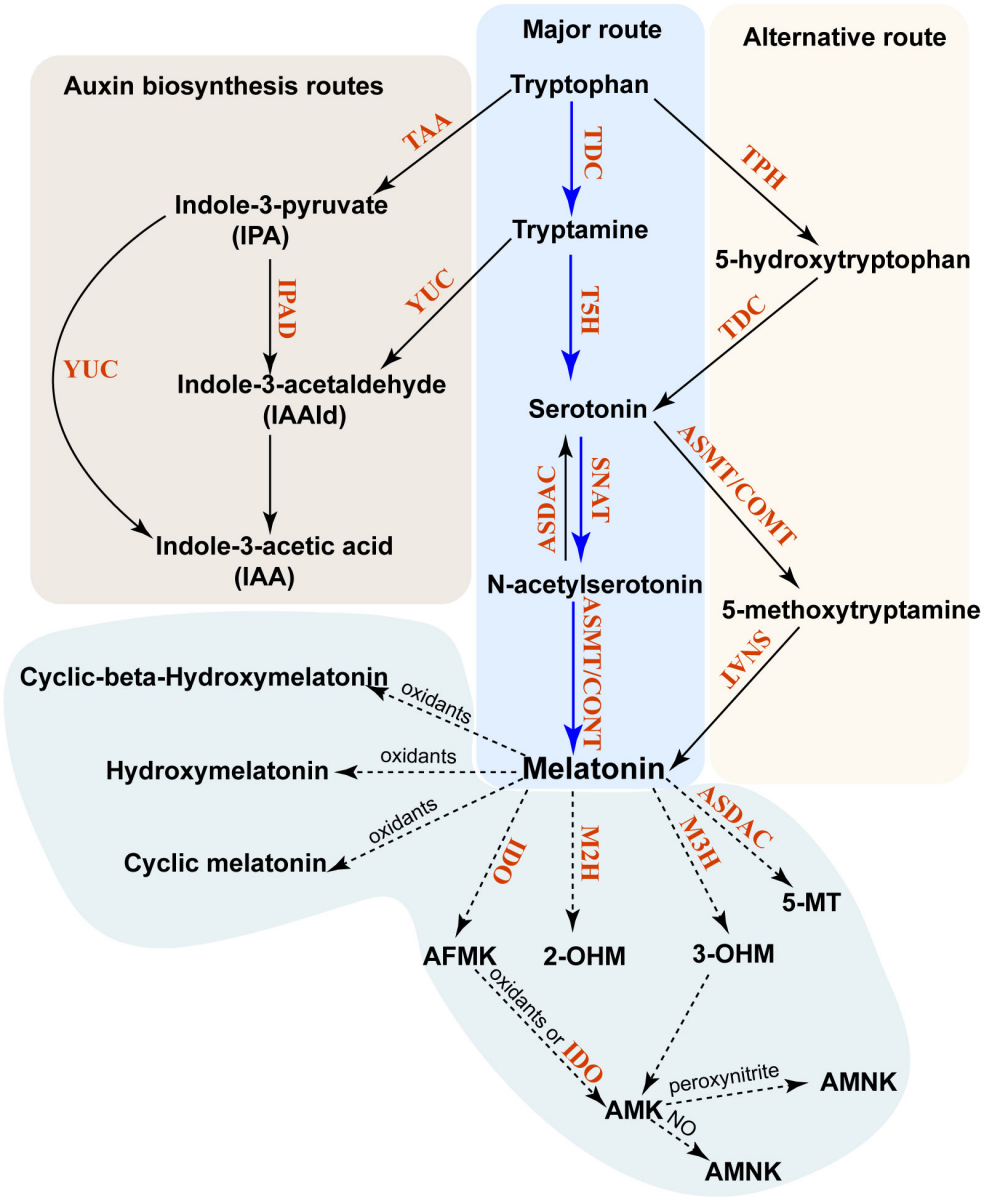
Biosynthesis and metabolism of phytomelatonin in plants. The major pathway of phytomelatonin synthesis is indicated by *solid blue arrows*, whereas the alternative pathway is indicated by *solid black arrows*. The phytomelatonin metabolism pathway is marked by *dashed arrows*. All enzymes in these pathways are indicated in *red*. *TDC*, tryptophan decarboxylase; *TPH*, tryptophan-5-hydroxylase; *T5H*, tryptamine-5-hydroxylase; *SNAT*, serotonin *N*-acetyltransferase; *AANAT*, aralkylamine-*N*-acetyltransferase; *ASMT*, *N*-acetylserotonin methyltransferase; *COMT*, caffeic acid *O*-methyltransferase; *ASDAC*, *N*-acetylserotonin deacetylase; *AADC*, aromatic-l-amino acid decarboxylase; *M2H*, melatonin 2-hydroxylase; *M3H*, melatonin 3-hydroxylase; *AMK*, *N*-acetyl-5-methoxykynuramine; *AFMK*, *N* ^1^-acetyl-*N* ^2^-formyl-5-methoxyaminourea; *IDO*, indoleamine 2,3-dioxygenase; *2-OHM*, 2-hydroxymelatonin; *3-OHM*, cyclic 3-hydroxymelatonin.

Plants possess both a major biosynthetic pathway (MP) and an alternative biosynthetic pathway (AP) for phytomelatonin (Arnao et al., 2022). Phytomelatonin synthesis is initiated in the shikimic acid pathway, with carbon dioxide used as the initial reactant (Arnao et al., 2022). The MP uses the shikimic acid pathway-derived tryptophan as a precursor to produce tryptamine using tryptophan decarboxylase (TDC). Tryptamine is then converted to 5-HT (serotonin) in a hydroxylation reaction catalyzed by tryptamine 5-hydroxylase (T5H). In the final two steps, serotonin is acetylated by serotonin *N*-acetyltransferase (SNAT) to form *N*-acetylserotonin, which is then converted into phytomelatonin by *N*-acetylserotonin methyltransferase (ASMT) in aquatic algae or caffeic acid-3-*O*-methyltransferase (COMT) in land plants (Sun et al., 2021; Zhao et al., 2021b) (**Figure 1**). In the AP, the shikimic acid pathway-derived tryptophan is initially hydroxylated to 5-hydroxytryptophan by TPH and then decarboxylated to form serotonin by TDC. Similarly, serotonin can be catalyzed by ASMT or COMT to form 5-methoxytryptamine (5-MT), which is then converted into phytomelatonin by SNAT (Arnao and Hernández-Ruiz, 2014) (**Figure 1**). The MP is dominant under normal conditions, whereas the AP becomes dominant when plants are exposed to environmental stressors (Tan and Reiter, 2020).

Unlike in animals, where melatonin is metabolized into 4-hydroxymelatonin (4-OHM) and 6-hydroxymelatonin (6-OHM) by the cytochrome P450 enzyme, phytomelatonin can be metabolized into other metabolites in an enzymatic or other biochemical manner (Back, 2021). The predominant forms of these phytomelatonin metabolites are 2-hydroxymelatonin (2-OHM) and cyclic 3-hydroxymelatonin (3-OHM), which are catalyzed by melatonin 2-hydroxylase (M2H) and melatonin 3-hydroxylase (M3H), respectively (Byeon and Back, 2015; Lee et al., 2016). They belong to the 2-oxoglutarate-dependent dioxygenase (2-ODD) family (Byeon and Back, 2015; Lee et al., 2016).

Melatonin can also be converted into 2-OHM, 3-OHM, and 4-OHM without enzymes through their interactions with hydroxyl radicals (Back, 2021). Once 3-OHM is produced, it is further oxidized to *N* ^1^-acetyl-*N* ^2^-formyl-5-methoxykynuramine (AFMK) (de Almeida et al., 2003). Similarly, the interaction of phytomelatonin with H_2_O_2_ results in the oxidative cleavage of its indole ring and produces AFMK (Reiter et al., 2007). AFMK is then able to convert into *N*-acetyl-5-methoxykynuramine (AMK) with the help of electron/hydrogen-abstracting radicals or by reacting with indoleamine 2,3-dioxygenase (IDO) (Okazaki et al., 2010). This reaction will form *N* ^1^-acetyl-5-methoxy-3-nitrokynuramine (AMNK) in the presence of nitric oxide (NO) or 3-acetoamidome-thyl-6-methoxycinnolinone (AMMC) in the presence of peroxynitrite (Reiter et al., 2007; Okazaki et al., 2010). It has also been shown that phytomelatonin can be deacetylated into 5-MT by *N*-acetylserotonin deacetylase (ASDAC), which is also responsible for the conversion of *N*-acetylserotonin into serotonin (Lee et al., 2018).

From these pathways, the 2-OHM and 3-OHM that are synthesized in the chloroplasts and in the cytosol are the major products of phytomelatonin hydroxylation and are involved in senescence, seed germination, and flowering in *Arabidopsis* (Lee and Back, 2021; Lee and Back, 2022a; Lee and Back, 2022b). The phytomelatonin MP, as well as its metabolites, are present in multiple organelles or subcellular compartments, including the mitochondria (Wang et al., 2017), chloroplasts (Zheng et al., 2017), and the cytosol (Back et al., 2016). This subcellular compartmentalization of phytomelatonin in plants may have two key advantages. First, acetyl-CoA, which is a cofactor of phytomelatonin biosynthesis, is synthesized in the mitochondria and chloroplasts and may increase the biosynthetic efficiency of phytomelatonin as its synthetic enzymes are also located in these organelles. Second, the biosynthesis of phytomelatonin in the mitochondria and chloroplasts may protect them from excess ROS production in these organelles (Tan and Reiter, 2020).

In mammalian cells, oligopeptide transporters (i.e., PEPT1/2) have been shown to facilitate the transportation of melatonin into the mitochondria, where it induces cell apoptosis (Huo et al., 2017). Melatonin can also be transported into cells by glucose transporter 1 (GLUT1) (Hevia et al., 2015). Therefore, the presence of PEPT1/2 or GLUT1 in a specific organelle may allow precise control of the melatonin levels in order to mitigate excess ROS and RNS. Notably, the GLUT1 homologs (**Supplementary Figure S1**), as well as the glucose transportation transporters, are also present in some plant species, such as *Medicago truncatula*, *Solanum lycopersicum*, and *Solanum tuberosum* (Aluri and Büttner, 2007; Cho et al., 2011). Whether and how these GLUT1 homologs and transporters are involved in phytomelatonin signaling require more research in the future.

**Figure 2:**
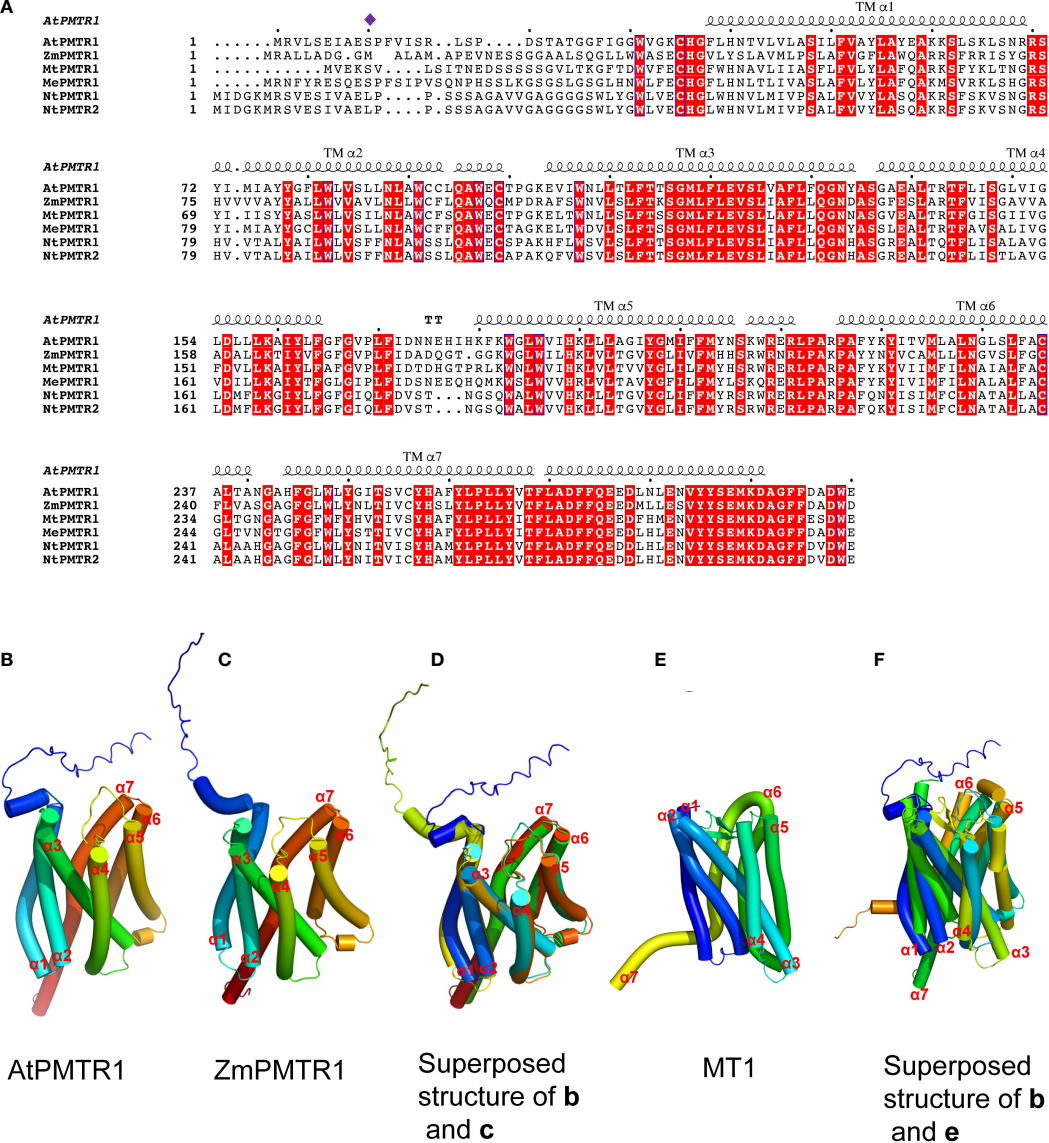
Multiple sequence alignment and structural modeling of AtPMTR1 and its homologs from *Medicago sativa* L. (MtPMTR1: XP_039685271.1), *Nicotiana benthamiana* (NtPMTR1: XP_019237084.1, NtPMTR2: XP_016433779.1), *Zea mays* (ZmPMTR1:NP_001136530.1), and cassava (MePMTR1: XP_021623418.1). **(A)** Multiple sequence alignment of AtPMTR1 and its homologs. Residues responsible for the binding of phytomelatonin are indicated by *purple diamonds*. Seven-transmembrane α-helices (TMα1–7) are marked on the sequence. **(B–F)** Structural modeling of PMTR1 from *A thaliana* and *Z. mays*. **(B, C)** Structure of AtPMTR1 from *A. thaliana* **(B)** and *Z. mays* **(C)** predicted with AlphaFold2 (https://alphafold.ebi.ac.uk). **(D)** Cylindrical presentation of superimposed AtPMTR1 and ZmPMTR1. **(E)** Structure of MT1 (PDB:6ME2, only 1–320 amino acids are displayed). **(F)** Cylindrical presentation of superimposed AtPMTR1 and MT1. Comparisons of the protein structures were performed using the TM-align server (https://zhanggroup.org/TM-align) (Zhang and Skolnick, 2005) and the resulting structures were visualized using PyMol software version 2.5.

In addition to phytomelatonin synthesis, tryptophan and tryptamine pools within plant cells are also utilized to synthesize indole-3-acetic acid (IAA) (Mashiguchi et al., 2011; Won et al., 2011). Tryptophan can be converted into indole-3-pyruvate (IPA) by tryptophan aminotransferase in *Arabidopsis* (TAA) (Won et al., 2011). Consequently, the IPA produced can then be converted into either IAA by YUCCA (YUC), which is a flavin monooxygenase, or into indole-3-acetaldehyde (IAAld) by IPA decarboxylase (IPD) (Mashiguchi et al., 2011; Won et al., 2011). Moreover, the tryptamine produced from tryptophan can also be converted into IAAld by YUC (Won et al., 2011) (**Figure 2**).

## Phytomelatonin receptor PMTR1 and its homologs identified in plants

In 2018, the Chen Lab discovered the first phytomelatonin receptor, AtPMTR1, in the model plant *A. thaliana*, which has been considered as a turning point in phytomelatonin research (Arnao and Hernández-Ruiz, 2019; Li et al., 2022). AtPMTR1 is thought to be a seven-transmembrane protein that interacts with the GPA1 in *Arabidopsis*, which activates NADPH oxidase-mediated H_2_O_2_ production, increases Ca^2+^ influx, and promotes K^+^ efflux, leading to stomatal closure (Wei et al., 2018). The amino acid sequences of AtPMTR1 and MT1/MT2 have a few similarities (Wei et al., 2018); furthermore, the three-dimensional structures of these receptors also exhibit some similarities (**Figure 2**), which indicates that MT1/MT2 and AtPMTR1 probably have descended through convergent rather than divergent evolution (Chen et al., 2022).

Lee and Back (2020) used tobacco plants and found that AtPMTR1 is localized in the cytoplasm (Lee and Back, 2020). In their study, the authors also found that AtPMTR1 is not a bona fide phytomelatonin receptor since several melatonin responsive genes could be induced by melatonin in *pmtr1* mutant lines (Lee and Back, 2020). However, more recent research has shown that the PMTR1 homologs act as membrane proteins and are required to perceive phytomelatonin signaling in the growth and stress response in a variety of plants (Yu et al., 2021; Wang et al., 2021b; Bai et al., 2022). For example, the PMTR1 found in alfalfa (*Medicago sativa* L.), tobacco (*Nicotiana benthamiana*), maize (*Zea mays*), and cassava are localized in the plasma membrane and are required to perceive phytomelatonin signaling in salt, immunity, drought, and leaf senescence, respectively (Kong et al., 2021; Yu et al., 2021; Wang et al., 2021b; Bai et al., 2022).

In addition to AtPMTR1, ZmPMTR1 is another putative phytomelatonin receptor identified from maize that acts as a plasma membrane-localized protein and exhibits a high binding activity to melatonin (Wang et al., 2021b). It plays an important role in phytomelatonin-mediated osmotic and drought tolerance by regulating the stomatal aperture (Wang et al., 2021b). However, it is unclear whether ZmPMTR1 also interacts with the G protein α subunit in maize since it shares very similar three-dimensional structures with AtPMTR1, with seven trans-membrane helices (**Figure 2**). Notably, MePMTR1, which was identified in cassava, showed a similar binding activity to melatonin *in vitro*, as AtPMTR1 did (Bai et al., 2022). It was also shown that MePMTR1 interacts with and is dephosphorylated by MePP2C1 at the serine 11 residue (S11), which leads to a decrease in its ability to bind melatonin (Bai et al., 2022). On the other hand, ZmPMTR1 shows high affinity to melatonin, and it harbors methionine rather than the serine residue at the 11-amino acid position (**Figure 2**), which suggests that there might be other unidentified residues that regulate melatonin binding.

## PMTR1 regulates stomatal closure rhythms

Stomata are microscopic valves on the epidermis of plants that are composed of a pair of guard cells and several accessory cells (Chater et al., 2017). Stomatal movements open and close the valves to control the gas exchange between plants and the environment, respiration, and the invasion of pathogens (Chater et al., 2017). These movements are affected by many environmental factors and endogenous signals, including light intensity, air humidity, carbon dioxide concentration, drought stress, and the circadian rhythm. Guard cells integrate light and temperature signals to control the stomatal aperture, which allows plants to predict day-to-night environmental changes that regulate their growth, development, metabolism, and responses to environmental stimuli (Kostaki et al., 2020). Stomatal rhythm represents another typical behavior controlled by circadian rhythms. Stomatal opening in the morning is regulated by several components of the circadian transcription–translation feedback loops (TTFLs) (Hassidim et al., 2017). For example, the *Arabidopsis toc1-1* mutant shortens the stomatal conductance rhythm (Somers et al., 1998). In addition, *Arabidopsis CCA1-OX* plants (*CCA1-OX*) under a guard cell-specific promoter (GC) loses the circadian rhythm-mediated stomatal movement (Dodd et al., 2005; Hassidim et al., 2017). Altogether, this research shows that the stomata play a specific role in controlling stress responses through circadian rhythms.

Similar to plants, mammals also exhibit circadian changes to melatonin through the action of melatonin receptors. It was found that AtPMTR1-mediated phytomelatonin signaling regulates stomatal closure, which implies that phytomelatonin plays a role in controlling the circadian stomatal movements in plants (Li et al., 2020). Furthermore, the stomatal aperture could also be regulated by ROS signaling dynamics in guard cells (Song et al., 2014). The accumulation of ROS is considered one of the earliest hallmarks of stomatal closure (Sierla et al., 2016). Indeed, Li et al. (2020) found that phytomelatonin biosynthesis follows daily rhythms under a light/dark cycle in *Arabidopsis* and that the diurnal stomatal closure and the expression of ROS-related genes were significantly impaired in *pmtr1* mutants. Therefore, the daily rhythms of phytomelatonin signaling mediated by PMTR1 are necessary to maintain ROS dynamics. In addition, ROS signaling feedback promotes the expression of genes related to phytomelatonin signaling, and nocturnal stomatal closure is a rhythmic output of this signaling that allows plants to avoid excess water loss during nighttime, promoting drought tolerance (Li et al., 2020).

## PMTR1 regulates seed germination and seedling growth by interacting with the signaling of other phytohormones

Seed germination is the initial step in plant growth and development, which is then followed by imbibition and germination (Penfield, 2017). The strength of a plant’s germination ability determines the growth and development of its seedlings (Penfield, 2017). In addition, seed germination is a fragile stage during plant growth that is critical for seedling formation and is affected by various complex intrinsic signals (i.e., plant hormones) and environmental factors (i.e., water, temperature, and light) (Penfield, 2017).

Phytomelatonin has a potential role in the regulation of seed germination. Several studies have found that phytomelatonin affects seed germination in a dose-dependent manner (Lv et al., 2021). Low concentrations of phytomelatonin (10 μM) had no effect on the seed germination rate of *A. thaliana*, but high concentrations (500 and 1,000 μM) significantly inhibited seed germination (Lv et al., 2021). It was also found that the presence of auxin could alleviate the inhibition of seed germination caused by high concentrations of melatonin, which indicates that the interactions between auxin and phytomelatonin coordinate to regulate seed germination in *Arabidopsis* (Lv et al., 2021).

Furthermore, the regulation of phytomelatonin in seed germination and seedling growth has been shown to be associated with PMTR1 (Yin et al., 2022). Yin et al. (2022) found that PMTR1 regulates seed germination and seedling growth by regulating the homeostasis of abscisic acid (ABA) in *Arabidopsis* (Yin et al., 2022). In their work, exogenous phytomelatonin inhibited seedling growth by increasing the levels of ABA, which were associated with the upregulation of 9-*cis*-epoxycarotenoid dioxygenase (*NCED*) genes in the ABA biosynthetic pathway (Yin et al., 2022). During seed development, the *Arabidopsis PMTR1* knockout mutant had an elevated ABA accumulation peak, while the *PMTR1*-overexpressing seeds showed lower ABA accumulation peaks, which resulted in smaller seed size compared to the wild type (Yin et al., 2022). During seed germination, it was also found that the *PMTR1*-overexpressing lines accumulated higher ABA levels in imbibed seeds compared to dry seeds, while *PMTR1* knockouts showed lower levels of ABA after imbibition compared to the wild type (Yin et al., 2022). Therefore, the knockout of *PMTR1* caused faster germination, while overexpression caused slower germination (Yin et al., 2022). Together, these results indicate that PMTR1 is involved in seed development and seedling growth through cross talk with other phytohormones, such as ABA.

## Phytomelatonin inhibited leaf senescence using a PMTR1-dependent mechanism

Photosynthesis mainly occurs in plant leaves; however, senesce inhibits photosynthesis and directly affects crop productivity (Woo et al., 2019). To improve crop photosynthetic efficiency and productivity, it is necessary to delay premature leaf senescence. Typically, leaf senescence is an intricate and coordinated process that is characterized by the production of ROS and the breakdown of chloroplasts, which reduces the chlorophyll content and disrupts physiological functions and cell integrity, leading to programmed cell death (Kim et al., 2018; Zhao et al., 2021c). Senescence is not only controlled by endogenous developmental programs, such as age and circadian rhythms, but also by external environmental signals that include darkness, nutrient deficiency, high salinity, extreme temperature, drought, and pathogen infection (Woo et al., 2019; Zhao et al., 2021c).

Phytomelatonin has been shown to play a major role in leaf senescence by maintaining an intact leaf structure, eliminating ROS, ensuring high photosynthetic efficiency by inhibiting chlorophyll degradation, and accelerating chlorophyll *de novo* synthesis (Zhao et al., 2021c). In particular, Arnao and Hernández-Ruiz (2009) found that 1 mM melatonin could inhibit barley leaf senescence and delay chlorophyll degradation in the later stages of plant growth, possibly *via* interactions with ABA and 6-furfuryl-aminopurine (Arnao and Hernández-Ruiz, 2009). In ryegrass, phytomelatonin decreased the transcript levels of senescence-related genes (i.e., *IpS4G12.1* and *Lpl36*) and inhibited the expression of *IAA17*, *AXR3*, and *NAC*, which decreased the expression of *SEN4* and *S4G12* and delayed leaf senescence (Shi et al., 2015c). Moreover, phytomelatonin can delay senescence in apple leaves by inhibiting hydrolase activity during photosynthesis, as well as the redox responses from plastids in these leaves (Wang et al., 2012).

**Figure 3:**
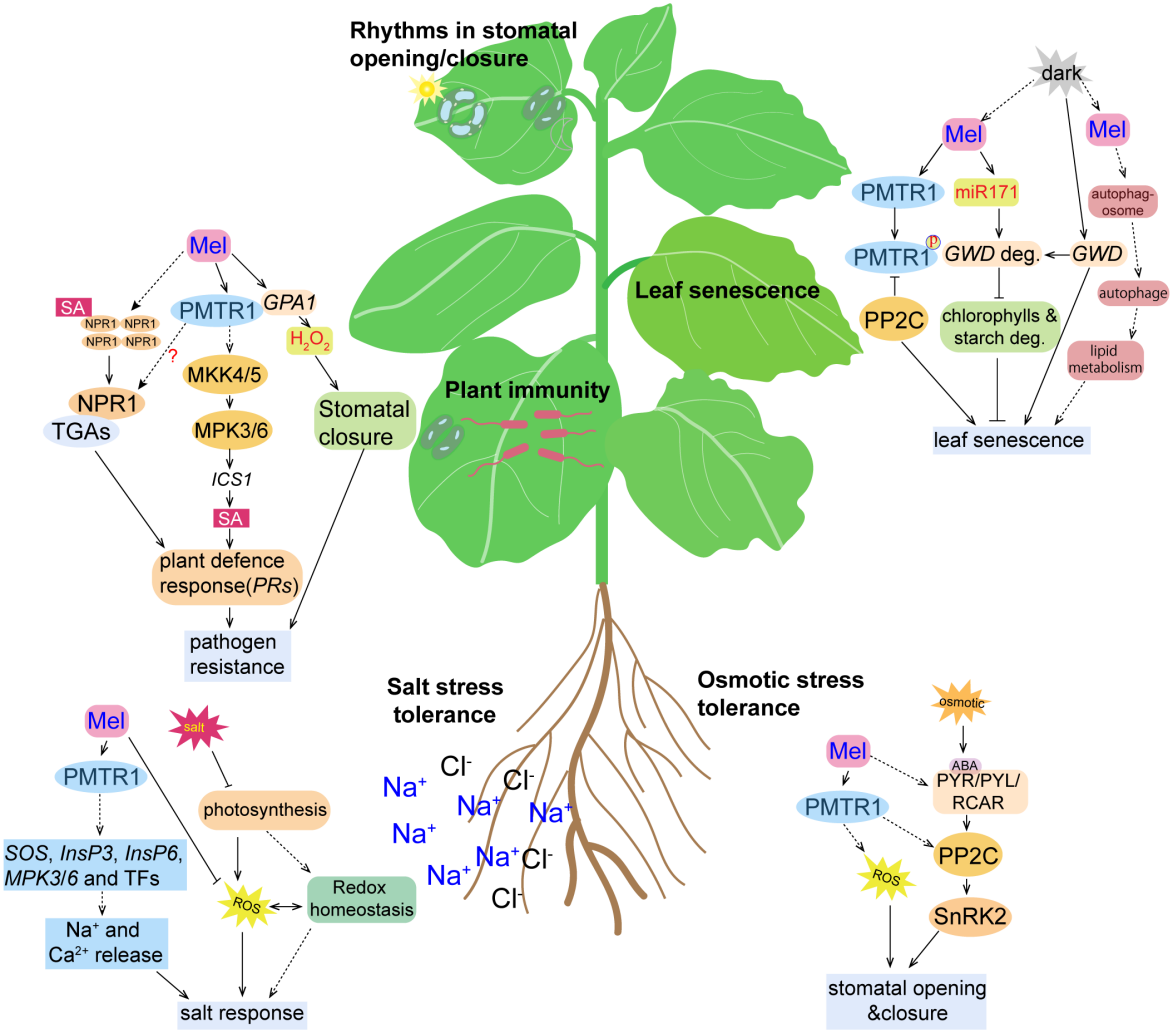
Cartoon illustration of the activity of phytomelatonin and the PMTR1-mediated signaling pathways under different stress conditions. High salinity results in the inhibition of photosynthesis, accumulation of reactive oxygen species (ROS), and the disruption of redox homeostasis, all of which were alleviated by phytomelatonin treatment. Under salt stress conditions, phytomelatonin is perceived by its PMTR1 receptor, which induces the expression of genes encoding *SOS*, *InsP3*, *InsP6*, *MPK3*, and *MPK6*, as well as the expression of other transcription factors, leading to the release of Na^+^ and Ca^2+^ and enhancing the plant responses to salt stress. Under osmotic conditions, the plant stress signaling hormone abscisic acid (ABA) is accumulated and binds to its receptor PYR/PYL/RCAR, which may, in turn, activate SnRK2s. The activated SnRK2s regulates the expression of ABA-responsive genes through ABA-responsive element (ABRE) binding protein/ABRE binding factor (AREB/ABF) transcription factors. Phytomelatonin likely regulates plant adaptation to osmotic stress through interactions with PP2C by using PMTR1 to activate ABA-dependent signaling pathways. Phytomelatonin can also induce stomatal closure to prevent water loss and enhance plants’ tolerance to osmotic stress. Upon pathogen infection, the PMTR1-mediated activation of MKK4/5 and the subsequent phosphorylation of MPK3/6 can result in the upregulation of genes encoding the salicylic acid (SA) pathway, which contribute to pathogen resistance. Phytomelatonin may also trigger the translocation of NPR1 from the cytosol to the nucleus, where it activates the expression of defense genes. Phytomelatonin can also mediate NADPH-dependent H_2_O_2_ production to close the stomata, thereby restricting pathogen infection. Upon darkness exposure, the expression of *GWD*, a gene responsible for starch degradation, and the accumulation of the GWD protein are induced, which promotes starch degradation. Phytomelatonin can inhibit leaf senescence by inducing the expression of *miR170*, which targets *GWD* for degradation. In dark conditions, the phytomelatonin signals can also be perceived by PMTR1 to initiate unknown downstream signaling to inhibit leaf senescence. PP2C can interact with and dephosphorylate PMTR1 to fine-tune phytomelatonin signaling. However, it can also interact with MeRAV1, MeRAV2, and MeWRKY20 to inhibit the transcriptional activation of *MeRAV1/2* and *MeWRKY20* on the phytomelatonin biosynthetic genes.

Apart from regulating photosynthesis, phytomelatonin also controls the metabolism of starches and lipids, and autophage in plants, which regulates dark-induced leaf senescence (**Figure 3**). For example, phytomelatonin was found to delay dark-induced leaf senescence by regulating the expression of *miR171b*, which targets and inhibits the expression of the key gene *GWD* (α-glucan water dikinase) that is responsible for starch degradation in tomatoes (Wang et al., 2022). In addition, phytomelatonin induces autophagy, which is required for lipid homeostasis in dark conditions (Barros et al., 2021). For example, the autophagy-associated proteins MeATG8b/8c/8e interact with the phytomelatonin biosynthesis enzymes MeTDC2, MeASMT2, and MeASMT3 in cassava (Wei et al., 2020). However, it is still unclear whether phytomelatonin controls dark-induced leaf senescence by affecting the autophagic activity or the lipid metabolism, which should be explored further in the future. Still, the phytomelatonin-mediated senescence process is complex and may involve complicated cross talk between multiple signaling pathways (i.e., phytohormones) (Zhao et al., 2021c).

Notably, it has also been found that phytomelatonin-regulated plant leaf senescence is dependent on the PMTR1 receptors (Bai et al., 2022). Bai et al. showed that, under dark conditions, 50 μM exogenous phytomelatonin treatment significantly delayed the senescence of cassava leaves, which resulted in greener leaves with higher contents of chlorophyll *a* and *b* (Bai et al., 2022). However, phytomelatonin was not able to delay leaf senescence in *MePMTR1*-silenced plants, which indicates that MePMTR1 is essential for phytomelatonin to delay dark-induced cassava senescence (Bai et al., 2022). Furthermore, MePP2C, a phosphatase protein, negatively regulated phytomelatonin signaling by interacting with the phytomelatonin metabolism-related transcription factors MeRAV1/2 and MeWRKY20 and the receptor *MePMTR1*. Here, the *MePP2C* gene-silenced plants exhibited a dark-induced leaf senescence-insensitive phenotype. However, cassava leaves co-silenced with *MePMTR1* and *MePP2C1* were more sensitive to dark-induced leaf senescence, which reversed the phenotype of the *MePP2C1*-silenced cassava leaves, and were not significantly different from the *MePMTR1*-silenced cassava in terms of dark-induced leaf senescence. Together, these results suggest that MePMTR1 is a downstream regulator of PP2C (Bai et al., 2022) (**Figure 3**).

## PMTR1 regulates plant innate immunity

Plants are also affected by a variety of pathogens during their growth and have evolved a complex and efficient innate immune system to fight the invasion of external pathogens. The plant immune receptors on the cell surface that are responsible for sensing microorganisms, such as host-derived molecular models of immunogenicity or pathogen- or microbe-associated molecular patterns (PAMPs/MAMPs), are known as pattern recognition receptors (PRRs) (Dodds and Rathjen, 2010). Plants have two layers of innate immunity, i.e., PRR-triggered immunity (PTI) and effector-triggered immunity (ETI), used to sense and fight pathogens (Jones and Dangl, 2006). Innate immunity is initiated by PAMPs/MAMPs through the activity of PRRs on the surface of plant cells (Bittel and Robatzek, 2007; Meng and Zhang, 2013).

It has long been shown that salicylic acid (SA) plays an essential role in basal defense and systemic acquired resistance (SAR) partly by promoting the closure of the stomata, which limits the entry of pathogens (Peng et al., 2021). Phytomelatonin plays an important role in protecting plants from bacterial, fungal, and viral infections (Zeng et al., 2022). For example, exogenous phytomelatonin effectively inhibited the activity of *Rhizoctonia solanacearum* and enhanced the resistance of apple trees to brown spot disease (Yin et al., 2013). It has also been found that *Pseudomonas fluorescens* (*Pst* DC3000) infection increased the concentration of endogenous phytomelatonin in *A. thaliana*, and exogenously applied phytomelatonin induced the production of defense signals, such as NO and SA, that are responsible for plant defense against *Pst* DC3000 infection (Shi et al., 2015a; Shi et al., 2015b). Similarly, phytomelatonin increased disease resistance against *Botrytis cinerea* in cherry tomato fruit and enhanced the resistance to tobacco mosaic virus (TMV) in *Solanum lycopersicum* by activating the SA pathway (Zhao et al., 2019b; Li et al., 2022).

In the biosynthetic pathways for phytomelatonin and SA, chorismic acid is shared as a common precursor (Hernández-Ruiz and Arnao, 2018). Interestingly, the amount of SA available induces defense genes including *PR1*, *ICS1*, and *PDF1.2*, which were found to decrease in *snat1* mutants compared to wild-type *Arabidopsis* during pathogen infection (Lee et al., 2015). Together, these results suggest that phytomelatonin acts upstream of SA to regulate its accumulation. Given that NPR1 (NONEXPRESSER OF PATHOGENESIS-RELATED GENES 1) acts as the receptor of SA that transduces downstream signals, it is likely that it translocates from the cytoplasm into the nucleus, where it activates the expression of the defense genes after phytomelatonin treatment (Lee et al., 2015). However, whether the regulation of NPR1 also depends on AtPMTR1 needs to be investigated further (**Figure 3**).

Notably, exogenous phytomelatonin treatments also induced the phosphorylation and activation of MAPK cascade signaling (Lee and Back, 2016; Yang et al., 2021). Specifically, phytomelatonin has been shown to activate MPK3 and MPK6, which are compromised in the MAPK kinase (MKK) *mkk4*, *mkk5*, *mkk7*, and *mkk9* mutants, as well as the *atpmtr1* mutant of *Arabidopsis.* Together, they are required to induce the expression of the defense-related genes, including isochorismate synthase 1 (*ICS1*), pathogenesis-related gene 1 (*PR1*), and pathogenesis-related gene 5 (*PR5*) (Lee and Back, 2016; Yang et al., 2021). However, it remains unknown how AtPMTR1 mediates the activation of MPK3/6.

In addition to CO_2_ assimilation for photosynthesis and water loss during respiration, the stomata are regarded as the main sites for foliar pathogen invasion. Plants have evolved a mechanism to close the stomata after sensing PAMPs/MAMPs in order to restrict pathogen invasion, known as stomatal defense or stomatal immunity (Melotto et al., 2017). Previously, Yang et al. (2021) found that phytomelatonin significantly enhanced the innate immunity of *Panax notoginseng* through ROS- and MAPK-mediated stomatal closure (Yang et al., 2021). They also found that phytomelatonin and flg22 (a bacterial elicitor peptide corresponding to the most conserved domain of flagellin) induced plant stomatal immunity and the expression of disease resistance genes that are dependent on PMTR1 (Yang et al., 2021). Furthermore, *pmtr1* mutant plants were also insensitive to flg22-induced stomatal immunity, which suggests that PMTR1 may be a downstream component of the FLS2 (Flagellin Sensing 2) and BAK1 (Brassinosteroid Insensitive 1-Associated Kinase 1) immune receptor signaling pathways (Yang et al., 2021). Kong et al. discovered two homologs of AtPMTR1 in tobacco: trP47363 and trP13076 (Kong et al., 2021). The results of their study showed that phytomelatonin and its analogs, 5-MT and 5-methoxyoctamine, regulated stomatal closure, ROS, and SA signaling and that the expression of disease resistance genes was dependent on trP47363 and trP13076 to enhance tobacco resistance to *Phytophthora* (Kong et al., 2021).

## PMTR1 is involved in drought stress

Drought is one of the most detrimental factors that influence plant growth, development, and productivity; thus, it has a significant impact on agricultural yields (Mushtaq et al., 2022). A prominent feature of drought stress is the reduction of water availability that typically leads to the limitation of photosynthesis, which changes stomatal conductance (Sharma and Zheng, 2019). Drought also induces the production of ROS and leads to oxidative cell damage. As a multifunctional molecule, phytomelatonin is capable of protecting plants from these adverse effects of drought stress by enhancing the ROS scavenging efficiency and maintaining redox homeostasis, thus protecting the photosynthetic apparatus (Sharma and Zheng, 2019; Tiwari et al., 2021; Mushtaq et al., 2022).

Other studies have also shown that the resistance of plants to drought stress is positively correlated with the accumulation and expression of antioxidant enzymes (Sharma and Zheng, 2019; Tiwari et al., 2021). Li et al. also found that phytomelatonin induced the expression of genes involved in ABA catabolism and enhanced the activities of antioxidant enzymes to indirectly detoxify ROS, which contributed to the drought tolerance of *Malus prunifolia* under stress conditions (Li et al., 2015). Furthermore, heterologous expression of the apple *MzSNAT5* gene in *Arabidopsis* can increase mitochondrial phytomelatonin synthesis, reduce oxidative damage, and improve plant drought tolerance (Wang et al., 2017). In addition, long-term irrigation with phytomelatonin can inhibit the expression of senescence-related genes (e.g., *SAG12* and *PAO*), which can improve the leaf photosynthetic efficiency, alleviate oxidative stress, and slow down drought-induced leaf senescence (Wang et al., 2013).

Previously, Ma et al. used wild-type and transgenic *Agrostis stolonifera* treated with exogenous phytomelatonin and found that drought-responsive genes (i.e., *JUB1* and *DREB2A*) were upregulated, while chlorophyll catabolism genes (i.e., *Chlase*, *PPH*, and *Chl-PRX*) were significantly downregulated (Ma et al., 2018). In addition, phytomelatonin synthesis-related genes (i.e., *TDC1*, *SNAT1*, and *COMT*) were significantly upregulated (Ma et al., 2018). It was also found that exogenous treatment with 100 mM phytomelatonin improved the growth, yield, and quality of *Moringa oleifera* by improving its photosynthetic pigments under drought stress (Sadak et al., 2020). Furthermore, phytomelatonin treatment in wheat under drought stress affected the expression of glycolytic proteins and activated the autophagy-related metabolic cascades (Cui et al., 2018). Therefore, phytomelatonin is likely to inhibit the degradation of chlorophyll and maintain photosynthetic efficiency, which alleviate the negative effects of drought stress and help plants adapt to arid environments.

Other studies have also found that PMTR1 is involved in plant drought stress by promoting stomatal closure (Li et al., 2020; Wang et al., 2021b). For example, Li et al. (2020) found that *Arabidopsis pmtr1* mutants lost the phenotype of stomatal rhythm closure, and the rate of water loss at night was significantly higher than that of wild-type and *AtPMTR1*-overexpressing transgenic plants (Li et al., 2020). Similarly, *ZmPMTR1* is required for plant tolerance to drought stress in maize (Wang et al., 2021b). Specifically, Wang et al. found that the disruption of *ZmPMTR1* increased the rate of water loss, whereas the heterologous expression of *ZmPMTR1* in *Arabidopsis pmtr1* mutants complemented their osmotic stress-sensitive phenotypes and phytomelatonin-induced stomatal closure (Wang et al., 2021b). It was also found through thermal infrared imaging experiments that the leaf temperature in a maize *Zmpmtr1* mutant under drought stress was lower than that in the wild type and was more sensitive to drought stress, which indicates that ZmPMTR1 is required for maize drought stress response and tolerance (Wang et al., 2021b). Altogether, these results suggest that PMTR1-mediated phytomelatonin signaling is probably conserved between monocot and dicot species for drought tolerance.

## PMTR1 involvement in salt stress resistance

Soil salinization is one of the most serious environmental stressors that affect crop production (Gong et al., 2020). Approximately 20% of the world’s 230 million hectares of irrigated farmland is salinized to varying degrees due to inappropriate irrigation and fertilization (Zhu, 2001). Salt stress affects plant growth and development through osmotic, ionic, and oxidative stress (Gong et al., 2020). Phytomelatonin protects plants from salt stress by regulating photosynthesis and ROS and ABA signaling (Zhan et al., 2019; Khan et al., 2022b). Under salt stress, phytomelatonin treatment reduces the net photosynthetic rate, as well as the photosystem II (PSII) maximum quantum efficiency (*F*_v_/*F*_m_) (Zhan et al., 2019). It also reduces total chlorophyll by inhibiting the stomatal closure of seedlings, which improves light energy absorption and electron transfer (Zhan et al., 2019).

Seed germination is a key life stage in plants, and salt stress is extremely detrimental to this stage (Ibrahim, 2016) since excessive accumulation of Na^+^ leads to oxidative stress-induced membrane damage (Isayenkov and Maathuis, 2019). Phytomelatonin has been shown to have beneficial effects on seed germination under salt stress. Chen et al. found that salt stress inhibited the germination of cotton seeds, and exogenous phytomelatonin increased the antioxidant enzyme activity of seeds and slowed the inhibitory effect of salt stress on cotton seed germination (Chen et al., 2020). Similarly, phytomelatonin was found to alleviate the inhibitory effect of salt stress on seed germination by enhancing the expression of *GA20ox* and *GA3ox*, thereby accelerating the synthesis of GA4 in ‘Starkrimson’ pear (*Pyrus communis* L.) (Gao et al., 2022).

The expression of MAPK genes (*MPK3*, *MPK4*, and *MPK6*) was induced by phytomelatonin during salt stress in cucumbers (Zhang et al., 2020). Phytomelatonin also promoted the synthesis of inositol-1,4,5-triphosphate (InsP3) and inositol hexaphosphate (InsP6), as well as the release of Ca^2+^, by regulating the phosphatidylinositol signaling system, which improved salt tolerance in cotton seedlings (Zhang et al., 2021). Notably, the induction of both Ca^2+^ influx and K^+^ efflux, as well as the phosphorylation of MPK3/6 by phytomelatonin in *Arabidopsis* was dependent on AtPMTR1 (Wei et al., 2018; Yang et al., 2021). Similarly, Yu et al. found that alfafa possesses MsPMTR1, which shares 71% homology with AtPMTR1 (**Figure 2**), and that the seedling mortality of *Mspmtr1* mutants was 35% higher than that of the wild type; moreover, the plant height and root length were reduced by 35% and 27% under salt stress, respectively (Yu et al., 2021). However, phytomelatonin treatment failed to restore the growth inhibition in *MsPMTR1*-silenced plants under salt stress (Yu et al., 2021), which suggests that phytomelatonin probably acts with the membrane-localized MsPMTR1 to improve salt stress tolerance (**Figure 3**). Together, these results suggest that, in response to salinity stress, phytomelatonin regulates downstream signaling cascades through PMTR1–MAPK signaling pathways to activate the expression of salt stress-responsive genes (**Figure 3**).

## PMTR1 improves plant osmotic stress tolerance

Like other abiotic stressors, water deficit, which results in osmotic stress, seriously inhibits the growth and development of plants, resulting in huge losses of crops worldwide (Gong et al., 2020). Drought and high salinity are the major causes of osmotic stress in plants (Xiong and Zhu, 2002). Specifically, osmotic stress has a mechanical impact on the cell wall, which increases cytosolic Ca^2+^ and other secondary signals, such as ROS and ABA synthesis (Gong et al., 2020). Osmotic stress-mediated gene expression is typically regulated by ABA-dependent and ABA-independent signaling pathways (Yoshida et al., 2014). Phytomelatonin can enhance the capacity of plants to scavenge ROS, which improves their resistance to osmotic stress (**Figure 3**). In addition, plants’ osmotic stress response and tolerance are regulated by phytomelatonin and involve the PMTR1 receptor (Wang et al., 2021a; Wang et al., 2021b). For example, osmotic stress significantly increased the transcription level of PMTR1 in wild-type plants in a mannitol dose-dependent manner (Wang et al., 2021a). Normally, wild-type plants and *pmtr1* mutants have similar taproot lengths and fresh weights; however, the *pmtr1* mutants exhibited higher osmotic sensitivity, shorter root systems, and lower fresh weights under osmotic stress (Wang et al., 2021a).

In phytomelatonin, the carbonyl and 5-methoxy groups most likely act as antioxidants that scavenge ROS (Tan et al., 2002). Phytomelatonin treatment also reduced the inhibitory effect of osmotic stress in soybean and increased the expression of PSI- and PSII-related genes (Zhang et al., 2019). Similarly, phytomelatonin regulated K^+^ homeostasis in rice and increased the resistance to salt stress through NADPH oxidase (Liu et al., 2020). In *Arabidopsis*, Wei et al. (2018) found that phytomelatonin was not able to induce stomatal closure and ROS production in NADPH oxidase gene mutants, such as *rbohC* and *rbohD/F* (Wei et al., 2018), which suggests that phytomelatonin-induced stomatal closure is dependent on NADPH oxidase-mediated ROS production. However, whether phytomelatonin-induced stomatal closure is also involved in resistance to osmotic stress needs further investigation.

Similar to the classical model of ABA regulation for stomatal closure, it was found that *Arabidopsis gpa1* mutants were also insensitive to phytomelatonin-induced stomatal closure (Wei et al., 2018). In *Arabidopsis* and *Vicia faba* L. (faba bean), phytomelatonin showed similar function in stomatal closure and signaling to ABA. These signaling processes include the GPA1-mediated activation of NADPH oxidase-dependent ROS production, Ca^2+^ transient influx, and cascades that are activated by MAPK (Wei et al., 2018; Yang et al., 2021). However, unlike ABA, which can promote stomatal closure over a wide range of concentrations, the concentration range of phytomelatonin that induces stomatal closure is limited (5–20 μM) (Wei et al., 2018). Furthermore, ABA can induce stomatal closure under light and can inhibit the opening of a closed stomata, while phytomelatonin only promotes stomatal closure and does not affect stomatal opening during light exposure (Wei et al., 2018). It has also been found that *pmtr1* mutants lost circadian stomatal closure, but remained sensitive to ABA-induced stomatal closure. Given that phytomelatonin affects ABA metabolism and that ABA is known to regulate osmotic stress response (Yoshida et al., 2014; Zhang et al., 2014; Li et al., 2015), their cross talk needs to be further explored in the future to understand how they mediate osmotic stress.

## Conclusion and prospectives

Phytomelatonin is widely present in plants and functions throughout their entire growth and development stages. In *Arabidopsis*, AtPMTR1 was the first putative phytomelatonin receptor discovered in plants that regulates stomatal closure. More recent related studies have shown that phytomelatonin-mediated seed germination and seedling growth, leaf senescence, and resistance to several biotic and abiotic stresses are dependent on PMTR1. Therefore, PMTR1-mediated phytomelatonin signaling may play varied roles in plant growth, development, and stress resistance.

However, there are still two overarching scientific issues that need further investigation in future work, as follows:

1. PMTR1-mediated signaling pathways and regulatory mechanisms. At present, only GPA1 and MAPK signaling members and signaling molecules, such as ROS and Ca^2+^, have been discovered, but their regulatory mechanisms are still not fully understood. In the future, research will need to focus on the mechanisms that link PMTR1 receptors and MAPK cascades in order to illustrate how activated and phosphorylated MPK3/6 are released when phytomelatonin signaling stops. Moreover, it remains unclear how PMTR1 recognizes and transduces phytomelatonin signals. Determination of the crystal structure of PMTR1 will shed light on the interactions between PMTR1 and its ligands. Furthermore, future work should also explore whether there are other receptors that mediate phytomelatonin signaling. Ultimately, the identification of other players that interact with PMTR1 and the components that integrate phytomelatonin signaling with other plant hormones should be carried using quantitative proteomics and liquid chromatography–tandem mass spectrometry analysis, which will greatly enhance our understanding of the cross talk between phytomelatonin and other signals.
2. The application of PMTR1 in agricultural production and crop breeding. Existing studies have shown that phytomelatonin plays an important role in plant growth, development, stress response, and many other functions that are dependent on the PMTR1 receptor. Therefore, changing the expression levels of *PMTR1* in crops through genetic modification and molecular breeding should be explored to improve the stress resistance of crops.

## Author contributions

QC and XP conceived this study. DK, NC, and XP wrote the manuscript. WZ, LL, and XP analyzed the data. XP, MG, and QC revised the manuscript. All authors contributed to the article and approved the submitted version.

## References

[B1] Aluri S. Büttner M. (2007). Identification and functional expression of the arabidopsis thaliana vacuolar glucose transporter 1 and its role in seed germination and flowering. Proc. Natl. Acad. Sci. 104 (7), 2537–2542. doi: 10.1073/pnas.0610278104 17284600 PMC1892959

[B2] Arnao M. Hernández-Ruiz J. (2009). Protective effect of melatonin against chlorophyll degradation during the senescence of barley leaves. J. Pineal. Res. 46 (1), 58–63. doi: 10.1111/j.1600-079X.2008.00625.x 18691358

[B3] Arnao M. B. Hernández-Ruiz J. (2014). Melatonin: plant growth regulator and/or biostimulator during stress? Trends Plant Sci. 19 (12), 789–797. doi: 10.1016/j.tplants.2014.07.006 25156541

[B4] Arnao M. B. Hernández-Ruiz J. (2019). Melatonin: A New Plant Hormone and/or a Plant Master Regulator? Trends Plant Sci. 24 (1), 38–48. doi: 10.1016/j.tplants.2018.10.010 30446305

[B5] Arnao M. B. Cano A. Hernández-Ruiz J. (2022). Phytomelatonin: an unexpected molecule with amazing performances in plants. J. Exp. Bot. 73 (17), 5779–5800. doi: 10.1093/jxb/erac009 35029657

[B6] Back K. (2021). Melatonin metabolism, signaling and possible roles in plants. Plant J. 105 (2), 376–391. doi: 10.1111/tpj.14915 32645752

[B7] Back K. Tan D.-X. Reiter R. J. (2016). Melatonin biosynthesis in plants: multiple pathways catalyze tryptophan to melatonin in the cytoplasm or chloroplasts. J. Pineal. Res. 61 (4), 426–437. doi: 10.1111/jpi.12364 27600803

[B8] Bai Y. Wei Y. Yin H. Hu W. Cheng X. Guo J.. (2022). PP2C1 fine-tunes melatonin biosynthesis and phytomelatonin receptor PMTR1 binding to melatonin in cassava. J. Pineal. Res. 73 (1), e12804. doi: 10.1111/jpi.12804 35488179

[B9] Barros J. A. S. Magen S. Lapidot-Cohen T. Rosental L. Brotman Y. Araujo W. L.. (2021). Autophagy is required for lipid homeostasis during dark-induced senescence. Plant Physiol. 185 (4), 1542–1558. doi: 10.1093/plphys/kiaa120 33793926 PMC8133563

[B10] Bittel P. Robatzek S. (2007). Microbe-associated molecular patterns (MAMPs) probe plant immunity. Curr. Opin. Plant Biol. 10 (4), 335–341. doi: 10.1016/j.pbi.2007.04.021 17652011

[B11] Byeon Y. Back K. (2015). Molecular cloning of melatonin 2-hydroxylase responsible for 2-hydroxymelatonin production in rice (Oryza sativa). J. Pineal. Res. 58 (3), 343–351. doi: 10.1111/jpi.12220 25728912

[B12] Chater C. C. C. Caine R. S. Fleming A. J. Gray J. E. (2017). Origins and evolution of stomatal development. Plant Physiol. 174 (2), 624–638. doi: 10.1104/pp.17.00183 28356502 PMC5462063

[B13] Chen L. Liu L. Lu B. Ma T. Jiang D. Li J.. (2020). Exogenous melatonin promotes seed germination and osmotic regulation under salt stress in cotton (Gossypium hirsutum l.). PloS One 15 (1), e0228241. doi: 10.1371/journal.pone.0228241 32004326 PMC6994006

[B14] Chen Q. Hou S. Pu X. Li X. Li R. Yang Q.. (2022). Secrets of phytomelatonin: Possible roles in darkness. J. Exp. Bot. 73 (17), 5828–5839. doi: 10.1093/jxb/erac168 35522068

[B15] Cho M. H. Lim H. Shin D. H. Jeon J. S. Bhoo S. H. Park Y. I.. (2011). Role of the plastidic glucose translocator in the export of starch degradation products from the chloroplasts in arabidopsis thaliana. New Phytol. 190 (1), 101–112. doi: 10.1111/j.1469-8137.2010.03580.x 21175634

[B16] Cui G. Sun F. Gao X. Xie K. Zhang C. Liu S.. (2018). Proteomic analysis of melatonin-mediated osmotic tolerance by improving energy metabolism and autophagy in wheat (Triticum aestivum l.). Planta 248 (1), 69–87. doi: 10.1007/s00425-018-2881-2 29564630

[B17] de Almeida E. A. Martinez G. R. Klitzke C. F. de Medeiros M. H. Mascio P. D. (2003). Oxidation of melatonin by singlet molecular oxygen (O2 (1Δg)) produces N1-acetyl-N2-formyl-5-methoxykynurenine. J. Pineal. Res. 35 (2), 131–137. doi: 10.1034/j.1600-079X.2003.00066.x 12887657

[B18] Dodd A. N. Salathia N. Hall A. Kévei E. Tóth R. Nagy F.. (2005). Plant circadian clocks increase photosynthesis, growth, survival, and competitive advantage. Science 309 (5734), 630–633. doi: 10.1126/science.1115581 16040710

[B19] Dodds P. N. Rathjen J. P. (2010). Plant immunity: towards an integrated view of plant–pathogen interactions. Nat. Rev. Genet. 11 (8), 539–548. doi: 10.1038/nrg2812 20585331

[B20] Dubbels R. Reiter R. Klenke E. Goebel A. Schnakenberg E. Ehlers C.. (1995). Melatonin in edible plants identified by radioimmunoassay and by high performance liquid chromatography-mass spectrometry. J. Pineal. Res. 18 (1), 28–31. doi: 10.1111/j.1600-079X.1995.tb00136.x 7776176

[B21] Ebisawa T. Karne S. Lerner M. R. Reppert S. M. (1994). Expression cloning of a high-affinity melatonin receptor from xenopus dermal melanophores. Proc. Natl. Acad. Sci. 91 (13), 6133–6137. doi: 10.1073/pnas.91.13.6133 7517042 PMC44152

[B22] Gao T. Liu X. Tan K. Zhang D. Zhu B. Ma F.. (2022). Introducing melatonin to the horticultural industry: physiological roles, potential applications, and challenges. Horticult. Res. 9, uhac094. doi: 10.1093/hr/uhac094 PMC9297156 35873728

[B23] Gong Z. Xiong L. Shi H. Yang S. Herrera-Estrella L. R. Xu G.. (2020). Plant abiotic stress response and nutrient use efficiency. Sci. China Life Sci. 63 (5), 635–674. doi: 10.1007/s11427-020-1683-x 32246404

[B24] Gouy M. Guindon S. Gascuel O. (2010). SeaView version 4: a multiplatform graphical user interface for sequence alignment and phylogenetic tree building. Mol. Biol. Evol. 27 (2), 221–224. doi: 10.1093/molbev/msp259 19854763

[B25] Hassidim M. Dakhiya Y. Turjeman A. Hussien D. Shor E. Anidjar A.. (2017). CIRCADIAN CLOCK ASSOCIATED1 (CCA1) and the circadian control of stomatal aperture. Plant Physiol. 175 (4), 1864–1877. doi: 10.1104/pp.17.01214 29084902 PMC5717738

[B26] Hattori A. Migitaka H. Iigo M. Itoh M. Yamamoto K. Ohtani-Kaneko R.. (1995). Identification of melatonin in plants and its effects on plasma melatonin levels and binding to melatonin receptors in vertebrates. Biochem. Mol. Biol. Int. 35 (3), 627–634. 7773197

[B27] Hernández-Ruiz J. Arnao M. B. (2018). Relationship of melatonin and salicylic acid in biotic/abiotic plant stress responses. Agronomy 8 (4), 33. doi: 10.3390/agronomy8040033

[B28] Hevia D. Gonzalez-Menendez P. Quiros-Gonzalez I. Miar A. Rodriguez-Garcia A. Tan D. X.. (2015). Melatonin uptake through glucose transporters: a new target for melatonin inhibition of cancer. J. Pineal. Res. 58 (2), 234–250. doi: 10.1111/jpi.12210 25612238

[B29] Huang X. Tanveer M. Min Y. Shabala S. (2022). Melatonin as a regulator of plant ionic homeostasis: implications for abiotic stress tolerance. J. Exp. Bot. 73 (17), 5886–5902. doi: 10.1093/jxb/erac224 35640481

[B30] Huo X. Wang C. Yu Z. Peng Y. Wang S. Feng S.. (2017). Human transporters, PEPT1/2, facilitate melatonin transportation into mitochondria of cancer cells: An implication of the therapeutic potential. J. Pineal. Res. 62, (4). doi: 10.1111/jpi.12390 28099762

[B31] Ibrahim E. A. (2016). Seed priming to alleviate salinity stress in germinating seeds. J. Plant Physiol. 192, 38–46. doi: 10.1016/j.jplph.2015.12.011 26812088

[B32] Isayenkov S. V. Maathuis F. J. (2019). Plant salinity stress: many unanswered questions remain. Front. Plant Sci. 10, 80. doi: 10.3389/fpls.2019.00080 30828339 PMC6384275

[B33] Johansson L. C. Stauch B. McCorvy J. D. Han G. W. Patel N. Huang X. P.. (2019). XFEL structures of the human MT2 melatonin receptor reveal the basis of subtype selectivity. Nature 569 (7755), 289–292. doi: 10.1038/s41586-019-1144-0 31019305 PMC6589158

[B34] Jones J. D. Dangl J. L. (2006). The plant immune system. nature 444 (7117), 323–329. doi: 10.1038/nature05286 17108957

[B35] Khan M. Ali S. Manghwar H. Saqib S. Ullah F. Ayaz A.. (2022a). Melatonin function and crosstalk with other phytohormones under normal and stressful conditions. Genes 13 (10), 1699. doi: 10.3390/genes13101699 36292584 PMC9602040

[B36] Khan T. A. Saleem M. Fariduddin Q. (2022b). Recent advances and mechanistic insights on melatonin-mediated salt stress signaling in plants. Plant Physiol. Biochem. 188, 97–107. doi: 10.1016/j.plaphy.2022.08.007 35995025

[B37] Kim J. Kim J. H. Lyu J. I. Woo H. R. Lim P. O. (2018). New insights into the regulation of leaf senescence in arabidopsis. J. Exp. Bot. 69 (4), 787–799. doi: 10.1093/jxb/erx287 28992051

[B38] Kong M. Sheng T. Liang J. Ali Q. Gu Q. Wu H.. (2021). Melatonin and its homologs induce immune responses *via* receptors trP47363-trP13076 in nicotiana benthamiana. Front. Plant Sci. 12, 1197. doi: 10.3389/fpls.2021.691835 PMC8278317 34276740

[B39] Kostaki K. I. Coupel-Ledru A. Bonnell V. C. Gustavsson M. Sun P. McLaughlin F. J.. (2020). Guard cells integrate light and temperature signals to control stomatal aperture. Plant Physiol. 182 (3), 1404–1419. doi: 10.1104/pp.19.01528 31949030 PMC7054865

[B40] Lee H. Y. Back K. (2016). Mitogen-activated protein kinase pathways are required for melatonin-mediated defense responses in plants. J. Pineal. Res. 60 (3), 327–335. doi: 10.1111/jpi.12314 26927635

[B41] Lee H. Y. Back K. (2020). The phytomelatonin receptor (PMRT1) arabidopsis Cand2 is not a bona fide G protein–coupled melatonin receptor. Melatonin. Res. 3 (2), 177–186. doi: 10.32794/mr11250055

[B42] Lee H. Y. Back K. (2021). 2-hydroxymelatonin, rather than melatonin, is responsible for RBOH-dependent reactive oxygen species production leading to premature senescence in plants. Antioxid. (Basel). 10, (11). doi: 10.3390/antiox10111728 PMC8614918 34829600

[B43] Lee H. Y. Back K. (2022a). 2-hydroxymelatonin promotes seed germination by increasing reactive oxygen species production and gibberellin synthesis in arabidopsis thaliana. Antioxid. (Basel). 11, (4). doi: 10.3390/antiox11040737 PMC9028592 35453427

[B44] Lee H. Y. Back K. (2022b). The antioxidant cyclic 3-hydroxymelatonin promotes the growth and flowering of arabidopsis thaliana. Antioxid. (Basel). 11, (6). doi: 10.3390/antiox11061157 PMC9219689 35740053

[B45] Lee H. Y. Byeon Y. Tan D. X. Reiter R. J. Back K. (2015). Arabidopsis serotonin n-acetyltransferase knockout mutant plants exhibit decreased melatonin and salicylic acid levels resulting in susceptibility to an avirulent pathogen. J. Pineal. Res. 58 (3), 291–299. doi: 10.1111/jpi.12214 25652756

[B46] Lee K. Lee H. Y. Back K. (2018). Rice histone deacetylase 10 and arabidopsis histone deacetylase 14 genes encode n-acetylserotonin deacetylase, which catalyzes conversion of n-acetylserotonin into serotonin, a reverse reaction for melatonin biosynthesis in plants. J. Pineal. Res. 64 (2), e12460. doi: 10.1111/jpi.12460 29247559

[B47] Lee K. Zawadzka A. Czarnocki Z. Reiter R. J. Back K. (2016). Molecular cloning of melatonin 3-hydroxylase and its production of cyclic 3-hydroxymelatonin in rice (Oryza sativa). J. Pineal. Res. 61 (4), 470–478. doi: 10.1111/jpi.12361 27500558

[B48] Lerner A. B. Case J. D. Takahashi Y. Lee T. H. Mori W. (1958). Isolation of melatonin, the pineal gland factor that lightens melanocyteS1. J. Am. Chem. Soc. 80 (10), 2587–2587. doi: 10.1021/ja01543a060

[B49] Li C. Tan D.-X. Liang D. Chang C. Jia D. Ma F. (2015). Melatonin mediates the regulation of ABA metabolism, free-radical scavenging, and stomatal behaviour in two malus species under drought stress. J. Exp. Bot. 66 (3), 669–680. doi: 10.1093/jxb/eru476 25481689

[B50] Li D. Wei J. Peng Z. Ma W. Yang Q. Song Z.. (2020). Daily rhythms of phytomelatonin signaling modulate diurnal stomatal closure *via* regulating reactive oxygen species dynamics in arabidopsis. J. Pineal. Res. 68 (3), e12640. doi: 10.1111/jpi.12640 32064655

[B51] Li S. Huan C. Liu Y. Zheng X. Bi Y. (2022). Melatonin induces improved protection against botrytis cinerea in cherry tomato fruit by activating salicylic acid signaling pathway. Sci. Hortic. 304. doi: 10.1016/j.scienta.2022.111299

[B52] Liu J. Shabala S. Zhang J. Ma G. Chen D. Shabala L.. (2020). Melatonin improves rice salinity stress tolerance by NADPH oxidase-dependent control of the plasma membrane k+ transporters and k+ homeostasis. Plant. Cell Environ. 43 (11), 2591–2605. doi: 10.1111/pce.13759 32196121

[B53] Lv Y. Pan J. Wang H. Reiter R. J. Li X. Mou Z.. (2021). Melatonin inhibits seed germination by crosstalk with abscisic acid, gibberellin, and auxin in arabidopsis. J. Pineal. Res. 70 (4), e12736. doi: 10.1111/jpi.12736 33811388

[B54] Ma X. Zhang J. Burgess P. Rossi S. Huang B. (2018). Interactive effects of melatonin and cytokinin on alleviating drought-induced leaf senescence in creeping bentgrass (Agrostis stolonifera). Environ. Exp. Bot. 145, 1–11. doi: 10.1016/j.envexpbot.2017.10.010

[B55] Mashiguchi K. Tanaka K. Sakai T. Sugawara S. Kawaide H. Natsume M.. (2011). The main auxin biosynthesis pathway in arabidopsis. Proc. Natl. Acad. Sci. 108 (45), 18512–18517. doi: 10.1073/pnas.1108434108 22025724 PMC3215075

[B56] Melotto M. Zhang L. Oblessuc P. R. He S. Y. (2017). Stomatal defense a decade later. Plant Physiol. 174 (2), 561–571. doi: 10.1104/pp.16.01853 28341769 PMC5462020

[B57] Meng X. Zhang S. (2013). MAPK cascades in plant disease resistance signaling. Annu. Rev. Phytopathol. 51 (1), 245–266. doi: 10.1146/annurev-phyto-082712-102314 23663002

[B58] Mushtaq N. Iqbal S. Hayat F. Raziq A. Ayaz A. Zaman W. (2022). Melatonin in micro-tom tomato: Improved drought tolerance *via* the regulation of the photosynthetic apparatus, membrane stability, osmoprotectants, and root system. Life 12 (11), 1922. doi: 10.3390/life12111922 36431057 PMC9696799

[B59] Ng K. Y. Leong M. K. Liang H. Paxinos G. (2017). Melatonin receptors: distribution in mammalian brain and their respective putative functions. Brain Structure. Funct. 222 (7), 2921–2939. doi: 10.1007/s00429-017-1439-6 28478550

[B60] Okazaki M. Higuchi K. Aouini A. Ezura H. (2010). Lowering intercellular melatonin levels by transgenic analysis of indoleamine 2,3-dioxygenase from rice in tomato plants. J. Pineal. Res. 49 (3), 239–247. doi: 10.1111/j.1600-079X.2010.00788.x 20609074

[B61] Penfield S. (2017). Seed dormancy and germination. Curr. Biol. 27 (17), R874–R878. doi: 10.1016/j.cub.2017.05.050 28898656

[B62] Peng Y. Yang J. Li X. Zhang Y. (2021). Salicylic acid: Biosynthesis and signaling. Annu. Rev. Plant Biol. 72, 761–791. doi: 10.1146/annurev-arplant-081320-092855 33756096

[B63] Reiter R. D-x T. Terron M. Flores L. Czarnocki Z. (2007). Melatonin and its metabolites: new findings regarding their production and their radical scavenging actions. Acta Biochim. Polonica. 54 (1), 1–9. doi: 10.18388/abp.2007_3264 17351668

[B64] Reppert S. M. Godson C. Mahle C. D. Weaver D. R. Slaugenhaupt S. A. Gusella J. (1995). Molecular characterization of a second melatonin receptor expressed in human retina and brain: the Mel1b melatonin receptor. Proc. Natl. Acad. Sci. 92 (19), 8734–8738. doi: 10.1073/pnas.92.19.8734 7568007 PMC41041

[B65] Sadak M. S. Abdalla A. M. Abd Elhamid E. M. Ezzo M. (2020). Role of melatonin in improving growth, yield quantity and quality of moringa oleifera l. plant under drought stress. Bull. Natl. Res. Centre. 44 (1), 1–13.

[B66] Sharma A. Zheng B. (2019). Melatonin mediated regulation of drought stress: Physiological and molecular aspects. Plants 8 (7), 190. doi: 10.3390/plants8070190 31248005 PMC6681211

[B67] Shi H. Chen Y. Tan D. X. Reiter R. J. Chan Z. He C. (2015a). Melatonin induces nitric oxide and the potential mechanisms relate to innate immunity against bacterial pathogen infection in arabidopsis. J. Pineal. Res. 59 (1), 102–108. doi: 10.1111/jpi.12244 25960153

[B68] Shi H. Qian Y. Tan D. X. Reiter R. J. He C. (2015b). Melatonin induces the transcripts of CBF/DREB1s and their involvement in both abiotic and biotic stresses in arabidopsis. J. Pineal. Res. 59 (3), 334–342. doi: 10.1111/jpi.12262 26182834

[B69] Shi H. Reiter R. J. Tan D. X. Chan Z. (2015c). INDOLE-3-ACETIC ACID INDUCIBLE 17 positively modulates natural leaf senescence through melatonin-mediated pathway in arabidopsis. J. Pineal. Res. 58 (1), 26–33. doi: 10.1111/jpi.12188 25324183

[B70] Sierla M. Waszczak C. Vahisalu T. Kangasjärvi J. (2016). Reactive oxygen species in the regulation of stomatal movements. Plant Physiol. 171 (3), 1569–1580. doi: 10.1104/pp.16.00328 27208297 PMC4936562

[B71] Somers D. E. Webb A. Pearson M. Kay S. A. (1998). The short-period mutant, toc1-1, alters circadian clock regulation of multiple outputs throughout development in arabidopsis thaliana. Development 125 (3), 485–494. doi: 10.1242/dev.125.3.485 9425143

[B72] Song Y. Miao Y. Song C. P. (2014). Behind the scenes: the roles of reactive oxygen species in guard cells. New Phytol. 201 (4), 1121–1140. doi: 10.1111/nph.12565 24188383

[B73] Stauch B. Johansson L. C. McCorvy J. D. Patel N. Han G. W. Huang X.-P.. (2019). Structural basis of ligand recognition at the human MT1 melatonin receptor. Nature 569 (7755), 284–288. doi: 10.1038/s41586-019-1141-3 31019306 PMC6696938

[B74] Sun C. Liu L. Wang L. Li B. Jin C. Lin X. (2021). Melatonin: a master regulator of plant development and stress responses. J. Integr. Plant Biol. 63 (1), 126–145. doi: 10.1111/jipb.12993 32678945

[B75] Suofu Y. Li W. Jean-Alphonse F. G. Jia J. Khattar N. K. Li J.. (2017). Dual role of mitochondria in producing melatonin and driving GPCR signaling to block cytochrome c release. Proc. Natl. Acad. Sci. 114 (38), E7997–E8006. doi: 10.1073/pnas.1705768114 28874589 PMC5617277

[B76] Tan D. Reiter R. J. Manchester L. C. Yan M. El-Sawi M. Sainz R. M.. (2002). Chemical and physical properties and potential mechanisms: melatonin as a broad spectrum antioxidant and free radical scavenger. Curr. Topics. Med. Chem. 2 (2), 181–197. doi: 10.2174/1568026023394443 11899100

[B77] Tan D.-X. Reiter R. J. (2019). Mitochondria: the birth place, battle ground and the site of melatonin metabolism in cells. Melatonin. Res. 2 (1), 44–66. doi: 10.32794/mr11250011

[B78] Tan D.-X. Reiter R. J. (2020). An evolutionary view of melatonin synthesis and metabolism related to its biological functions in plants. J. Exp. Bot. 71 (16), 4677–4689. doi: 10.1093/jxb/eraa235 32413108

[B79] Tan D.-X. Zheng X. Kong J. Manchester L. C. Hardeland R. Kim S. J.. (2014). Fundamental issues related to the origin of melatonin and melatonin isomers during evolution: relation to their biological functions. Int. J. Mol. Sci. 15 (9), 15858–15890. doi: 10.3390/ijms150915858 25207599 PMC4200856

[B80] Tiwari R. K. Lal M. K. Kumar R. Chourasia K. N. Naga K. C. Kumar D.. (2021). Mechanistic insights on melatonin-mediated drought stress mitigation in plants. Physiol. Plant. 172 (2), 1212–1226. doi: 10.1111/ppl.13307 33305363

[B81] Vantassel D. Roberts N. Oenill S. (1995). “Melatonin from higher-plants-isolation and identification of n-acetyl 5-methoxytryptamine,” in Plant physiology, vol. 2. (ROCKVILLE, MD: AMER SOC PLANT PHYSIOLOGISTS 15501 MONONA DRIVE), 101–101. 20855.

[B82] Wang K. Cai S. Xing Q. Qi Z. Fotopoulos V. Yu J.. (2022). Melatonin delays dark-induced leaf senescence by inducing miR171b expression in tomato. J. Pineal. Res. 72 (3), e12792. doi: 10.1111/jpi.12792 35174545

[B83] Wang L. F. Li T.-T. Zhang Y. Guo J.-X. Lu K.-K. Liu W.-C. (2021a). CAND2/PMTR1 is required for melatonin-conferred osmotic stress tolerance in arabidopsis. Int. J. Mol. Sci. 22 (8), 4014. doi: 10.3390/ijms22084014 33924609 PMC8069227

[B84] Wang L. F. Lu K. K. Li T. T. Zhang Y. Guo J. X. Song R. F.. (2021b). Maize PHYTOMELATONIN RECEPTOR1 functions in plant osmotic and drought stress tolerance. J. Exp. Bot. 73 (17), 5961–5973 doi: 10.1093/jxb/erab553 34922349

[B85] Wang L. Feng C. Zheng X. Guo Y. Zhou F. Shan D.. (2017). Plant mitochondria synthesize melatonin and enhance the tolerance of plants to drought stress. J. Pineal. Res. 63 (3), e12429. doi: 10.1111/jpi.12429 28599069

[B86] Wang P. Yin L. Liang D. Li C. Ma F. Yue Z. (2012). Delayed senescence of apple leaves by exogenous melatonin treatment: toward regulating the ascorbate–glutathione cycle. J. Pineal. Res. 53 (1), 11–20. doi: 10.1111/j.1600-079X.2011.00966.x 21988707

[B87] Wang P. Sun X. Li C. Wei Z. Liang D. Ma F. (2013). Long-term exogenous application of melatonin delays drought-induced leaf senescence in apple. J. Pineal. Res. 54 (3), 292–302. doi: 10.1111/jpi.12017 23106234

[B88] Wei J. Li D. X. Zhang J. R. Shan C. Rengel Z. Song Z. B.. (2018). Phytomelatonin receptor PMTR 1-mediated signaling regulates stomatal closure in arabidopsis thaliana. J. Pineal. Res. 65 (2), e12500. doi: 10.1111/jpi.12500 29702752

[B89] Wei Y. Bai Y. Cheng X. Zhu B. Reiter R. J. Shi H. (2020). The dual roles of melatonin biosynthesis enzymes in the coordination of melatonin biosynthesis and autophagy in cassava. J. Pineal. Res. 69 (1), e12652. doi: 10.1111/jpi.12652 32201970

[B90] Won C. Shen X. Mashiguchi K. Zheng Z. Dai X. Cheng Y.. (2011). Conversion of tryptophan to indole-3-acetic acid by TRYPTOPHAN AMINOTRANSFERASES OF ARABIDOPSIS and YUCCAs in arabidopsis. Proc. Natl. Acad. Sci. 108 (45), 18518–18523. doi: 10.1073/pnas.1108436108 22025721 PMC3215067

[B91] Woo H. R. Kim H. J. Lim P. O. Nam H. G. (2019). Leaf senescence: systems and dynamics aspects. Annu. Rev. Plant Biol. 70, 347–376. doi: 10.1146/annurev-arplant-050718-095859 30811218

[B92] Xiong L. Zhu J. K. (2002). Molecular and genetic aspects of plant responses to osmotic stress. Plant. Cell Environ. 25 (2), 131–139. doi: 10.1046/j.1365-3040.2002.00782.x 11841658

[B93] Yang Q. Peng Z. Ma W. Zhang S. Hou S. Wei J.. (2021). Melatonin functions in priming of stomatal immunity in panax notoginseng and arabidopsis thaliana. Plant Physiol. 187 (4), 2837–2851. doi: 10.1093/plphys/kiab419 34618091 PMC8644721

[B94] Yin L. Wang P. Li M. Ke X. Li C. Liang D.. (2013). Exogenous melatonin improves m alus resistance to m arssonina apple blotch. J. Pineal. Res. 54 (4), 426–434. doi: 10.1111/jpi.12038 23356947

[B95] Yin X. Bai Y. L. Gong C. Song W. Wu Y. Ye T.. (2022). The phytomelatonin receptor PMTR1 regulates seed development and germination by modulating abscisic acid homeostasis in arabidopsis thaliana. J. Pineal. Res. 72 (4), e12797. doi: 10.1111/jpi.12797 35319134

[B96] Yoshida T. Mogami J. Yamaguchi-Shinozaki K. (2014). ABA-dependent and ABA-independent signaling in response to osmotic stress in plants. Curr. Opin. Plant Biol. 21, 133–139. doi: 10.1016/j.pbi.2014.07.009 25104049

[B97] Yu R. Zuo T. Diao P. Fu J. Fan Y. Wang Y.. (2021). Melatonin enhances seed germination and seedling growth of medicago sativa under salinity *via* a putative melatonin receptor MsPMTR1. Front. Plant Sci. 12. doi: 10.3389/fpls.2021.702875 PMC8418131 34490006

[B98] Zeng H. Bai Y. Wei Y. Reiter R. J. Shi H. (2022). Phytomelatonin as a central molecule in plant disease resistance. J. Exp. Bot. 73 (17), 5874–5885. doi: 10.1093/jxb/erac111 35298631

[B99] Zhan H. Nie X. Zhang T. Li S. Wang X. Du X.. (2019). Melatonin: A small molecule but important for salt stress tolerance in plants. Int. J. Mol. Sci. 20 (3). doi: 10.3390/ijms20030709 PMC6387279 30736409

[B100] Zhang H. J. Zhang N. Yang R. C. Wang L. Sun Q. Q. Li D. B.. (2014). Melatonin promotes seed germination under high salinity by regulating antioxidant systems, ABA and GA 4 interaction in cucumber (C ucumis sativus l.). J. Pineal. Res. 57 (3), 269–279. doi: 10.1111/jpi.12167 25112973

[B101] Zhang M. He S. Zhan Y. Qin B. Jin X. Wang M.. (2019). Exogenous melatonin reduces the inhibitory effect of osmotic stress on photosynthesis in soybean. PloS One 14 (12), e0226542. doi: 10.1371/journal.pone.0226542 31869357 PMC6927616

[B102] Zhang T. Shi Z. Zhang X. Zheng S. Wang J. Mo J. (2020). Alleviating effects of exogenous melatonin on salt stress in cucumber. Sci. Hortic. 262, 109070. doi: 10.1016/j.scienta.2019.109070

[B103] Zhang Y. Fan Y. Rui C. Zhang H. Xu N. Dai M.. (2021). Melatonin improves cotton salt tolerance by regulating ROS scavenging system and Ca 2+ signal transduction. Front. Plant Sci. 12, 693690. doi: 10.3389/fpls.2021.693690 34262587 PMC8273866

[B104] Zhang Y. Skolnick J. (2005). TM-align: a protein structure alignment algorithm based on the TM-score. Nucleic Acids Res. 33 (7), 2302–2309. doi: 10.1093/nar/gki524 15849316 PMC1084323

[B105] Zhao D. Wang H. Chen S. Yu D. Reiter R. J. (2021a). Phytomelatonin: an emerging regulator of plant biotic stress resistance. Trends Plant Sci. 26 (1), 70–82. doi: 10.1016/j.tplants.2020.08.009 32896490

[B106] Zhao D. Yao Z. Zhang J. Zhang R. Mou Z. Zhang X.. (2021b). Melatonin synthesis genes n-acetylserotonin methyltransferases evolved into caffeic acid O-methyltransferases and both assisted in plant terrestrialization. J. Pineal. Res. 71 (3), e12737. doi: 10.1111/jpi.12737 33844336

[B107] Zhao D. Yu Y. Shen Y. Liu Q. Zhao Z. Sharma R.. (2019a). Melatonin synthesis and function: evolutionary history in animals and plants. Front. Endocrinol. 10, 249. doi: 10.3389/fendo.2019.00249 PMC6481276 31057485

[B108] Zhao L. Chen L. Gu P. Zhan X. Zhang Y. Hou C.. (2019b). Exogenous application of melatonin improves plant resistance to virus infection. Plant Pathol. 68 (7), 1287–1295. doi: 10.1111/ppa.13057

[B109] Zhao Y.-Q. Zhang Z.-W. Chen Y.-E. Ding C.-B. Yuan S. Reiter R. J.. (2021c). Melatonin: A potential agent in delaying leaf senescence. Crit. Rev. Plant Sci. 40 (1), 1–22. doi: 10.1080/07352689.2020.1865637

[B110] Zheng X. Tan D. X. Allan A. C. Zuo B. Zhao Y. Reiter R. J.. (2017). Chloroplastic biosynthesis of melatonin and its involvement in protection of plants from salt stress. Sci. Rep. 7 (1), 1–12. doi: 10.1038/srep41236 28145449 PMC5286529

[B111] Zhu J. K. (2001). Plant salt tolerance. Trends Plant Sci. 6 (2), 66–71. doi: 10.1016/s1360-1385(00)01838-0 11173290

