# Supplementary material for: The role of phytomelatonin receptor 1-mediated signaling in plant growth and stress response
Source: Front Plant Sci. 2023 Mar 10;14:1142753. doi: 10.3389/fpls.2023.1142753 (PMC10036441; doi:10.3389/fpls.2023.1142753)
Supplement: Supplementary file 1 [file DataSheet_1.pdf]

```
                      1
4ZW9|GLUT1            ---------- ---------- ---------- ---------- ---------- ----------
pm005071g0050         --MAAFSCSQ PRALRCSLRR DHGALVIASA TGEHRQSQLA SSEGWLEPQG CDTASNHRYS
Soltu.DM.02G026040    MQASTFTVKG NTGFGLQNRR ------ILQG VSDLRSRNLA ---------- ----------
Medtr7g082270         ---------- ---------- ---------- ---------- ---------- ----------
Solyc02g086160        MQASTFTVKG NTAFGLQNRR ------ILQG VSDLRSRTLA ---------- ----------

                     61
4ZW9|GLUT1            ---------- ---------- ---------- ---------- ---------- ----------
pm005071g0050         IYSTCIPHGR RAAHVCPPQC QVGPQLSQTE SHSRMQSVSA KCSHYDSTPR ASPRPTLAES
Soltu.DM.02G026040    ---------- ---------- ---------- ---------- ---------- ----------
Medtr7g082270         ---------- ---------- ---------- ---------- ---------- ----------
Solyc02g086160        ---------- ---------- ---------- ---------- ---------- ----------

                    121
4ZW9|GLUT1            ---------- ---------- ---------- -------MHH HHHHHHHHSG DEVDAGSGHM
pm005071g0050         ARATRCNKCG GSSGSTSDSS NTGQELPSGS AVSAQHGRQL QWRSRDSNVG GSVHNESSSY
Soltu.DM.02G026040    GKSLRMTERN SCFGVSMDSA SMGIEL---- -------GRAR --RTVQSVFG SSAKARSHRV
Medtr7g082270         --------MS SCCGLRSGSP IMETEL---- ------TSSS RGRGFGSIFG STVKPRPTRF
Solyc02g086160        GKSLRM--TG SCFGVSMDSA SMGIEL---- ------GRAR --RTVQSVFG SSAKARSHRV

                    181
4ZW9|GLUT1            ---------- ---------- ---------- ---------- ---------- ----------
pm005071g0050         LLKAAGLASQ QAETIGAWVN SLLQQHLGGR GDQFKEAESH GRSKGESSRT GASLSMERAN
Soltu.DM.02G026040    ---------- ---------- ---------- ---------- -RAAGE---- ----------
Medtr7g082270         ---------- ---------- ---------- ---------- -QTSDE---- ----------
Solyc02g086160        ---------- ---------- ---------- ---------- -RAAGE---- ----------

                    241
4ZW9|GLUT1            ---------- ---------- ---------- ---------- ------GTQK VTP-------
pm005071g0050         MASNTMAGST LARLSGGAIH HGGGSFAEHG LSQRSSPMVG ATPYEWELQQ VLPPLAPDNI
Soltu.DM.02G026040    ---------- ---------- ---------- ---------- ------DIED AAPLKVQG--
Medtr7g082270         ---------- ---------- ---------- ---------- ------DVED LLPNKSPG--
Solyc02g086160        ---------- ---------- ---------- ---------- ------DIED AAPLKVQG--

                    301
4ZW9|GLUT1            ---------- ---------- ---------- ---------- -ALIFAITVA TIGSFQFGYN
pm005071g0050         VSNYAAAPTK QDLEQQQQQP LLPRPRVLQH SSGGPQRPPI TNLLLHVLVA CIGAYLYGYH
Soltu.DM.02G026040    ---------- ---------- ---------Q SSG------- -SVLPYVGVA CLGAILFGYH
Medtr7g082270         ---------- ---------- ---------R PSG------- -TVFPYVGVA CLGAFLFGYH
Solyc02g086160        ---------- ---------- ---------Q SSG------- -SVLPYVGVA CLAAILFGYH

                    361
4ZW9|GLUT1            TGVINAPEKI IKEFITKTLT DKGNAPPSEV LLTSLWSLSV AIFSVGGMIG SFSVGLFVNR
pm005071g0050         LSVANGP--- -LDTIAQELG FAGSAVAKGS VVSTFL---- ----VGAFLG CTISSTTADG
Soltu.DM.02G026040    LGVVNGA--- -LEYLAKDLG IAENTVIQGW IVSTVL---- ----AGAFVG SFTGGVLADK
Medtr7g082270         LGVVNGA--- -LEYLAKDLR IAQNTVLQGW IVSTLL---- ----AGATVG SFTGGALADK
Solyc02g086160        LGVVNGA--- -LEYLAKDLG IAENTVIQGW IVSTVL---- ----AGAFVG SFTGGALADK

                    421
4ZW9|GLUT1            FGRRNSMLIV NLLAVTGGCF MGLCKVAKSV EMLILGRLVI GLFCGLCTGF VPMYIGEISP
pm005071g0050         IGRRRSFQAT ALPILLGSC- --VSAMSSSL PAMLLGRFLV GVGLGLGSPL TSLYISEISP
Soltu.DM.02G026040    FGRTKTFILD AIPLSVGAF- --LCTTAQSV QAMIIGRLLT GIGIGISSAI VPLYISEISP
Medtr7g082270         FGRTRTFQLD AIPLAIGGF- --LCATAQSV QTMIVGRSLA GIGIGIASAI VPLYISEISP
Solyc02g086160        FGRTKTFILD AIPLSVGAF- --LCTTAQSV QAMIIGRLLT GIGIGISSAI VPLYISEISP

                    481
4ZW9|GLUT1            TALRGAFGTL NQLGIVVGIL VAQIFGLEFI LGSEELWPLL LGFTILPAIL QSAALPFCPE
pm005071g0050         AALRGTLNSL PQVAACVGIL LALLMGVQF- PELPLGWRGC FWCGAVPAAT LVFLSEYIAD
Soltu.DM.02G026040    TEIRGTLGTV NQLFICIGIL VALVVGLPL- SGNPSWWRTM FGLALIPSVL LAIGMAFSPE
Medtr7g082270         TEIRGALGSV NQLFICIGIL AALVAGLPL- EGNPTWWRTM FGIAIVPSIL LALGMAICPE
Solyc02g086160        TEIRGTLGTV NQLFICIGIL VALVAGLPL- SGNPSWWRTM FGLALIPSVL LAIGMVFSPE

                    541
4ZW9|GLUT1            SPRFLLINRK EEENAKQILQ RLWGTQDVSQ -------DIQ EMK------- ------DESA
pm005071g0050         SPRWLAQRGR WTE-AEKALE LLRGASSSST AALAARAELL DLKATSVHAD AAATDTTSAS
Soltu.DM.02G026040    SPRWLYQQGR ISE-AETSIK RLYGKEKVAE -------VMG DL-------- ------EASA
Medtr7g082270         SPRWLYQQGK ISE-AEKAIK TLYGKEIVAS -------VMQ DL-------- ------TAAS
Solyc02g086160        SPRWLYQQGR ISE-AETSIK RLYGKEKVAE -------VMG DL-------- ------EASA

                    601
4ZW9|GLUT1            RMSQEKQVTV LELFRVSSYR QPIIISIVLQ LSQQLSGINA VFYYSTGIFK DAGVQEPIYA
pm005071g0050         SSASSSEVSW SALFARP-YR RVVLLGCGLF MLQQLSGINS VFYFSSSIFK AAGVTSEFAA
Soltu.DM.02G026040    RGSSEPDAGW LDLFSSR-YR KVVSIGAAMF LLQQLAGINA VVYYSTAVFR SAGITSDVAA
Medtr7g082270         QGSSEPEAGW SELFSSR-YQ KVVSIGASLF LLQQFAGINA VVYYSTSVFR SAGISSDVAA
Solyc02g086160        QGSSEPDAGW LDLFSSR-YR KVVSIGAAMF FLQQLAGINA VVYYSTAVFR SAGITSDVAA
```

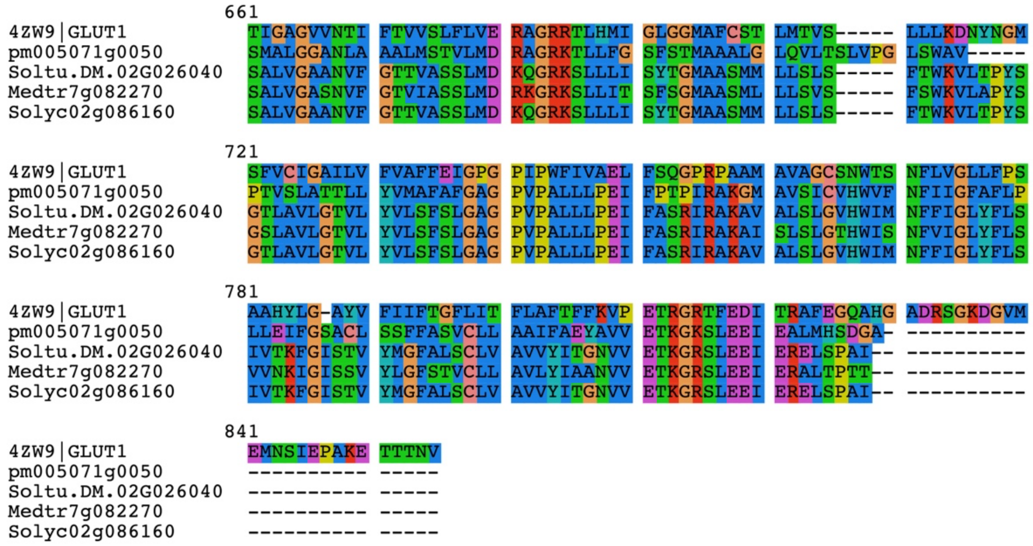


## SUPPLEMENTARY MATERIAL

**Figure S1 Multiple sequence alignment of GLUT1 in *Homo sapiens* and several plant species.** *H. sapiens* glucose transporter 1 (GLUT1, pdb accession number: 4ZW9) and the protein sequence of the GLUT1 homolog in *Penium margaritaceum* (Accession number: pm005071g0050) were retrieved from the Penium Genome Database (http://bioinfo.bti.cornell.edu/cgi-bin/Penium/home.cgi). GLUT1 homologs from other plant species including *Solanum lycopersicum* (Accession number: Solyc02g086160), *Solanum tuberosum* (Accession number: Soltu.DM.02G026040) and *Medicago truncatula* (Medtr7g082270) were retrieved from the Phytozome database (https://phytozome-next.jgi.doe.gov). Multiple sequence alignments of GLUT1 were performed using MUSCLE with default parameters using SeaView software Version 5.0.5 (Gouy et al. 2010).

### References


Gouy M, Guindon S, Gascuel O (2010) SeaView version 4: a multiplatform graphical user interface for sequence alignment and phylogenetic tree building. Molecular biology and evolution 27 (2):221-224
